# Limitations of the Glasgow Coma Scale: Challenges and Considerations

**DOI:** 10.7759/cureus.78900

**Published:** 2025-02-12

**Authors:** Christopher Andraos, Amman Siddiqi, James Brazdzionis, Javed Siddiqi

**Affiliations:** 1 Neurological Surgery, Arrowhead Regional Medical Center, Colton, USA; 2 Research, Arrowhead Regional Medical Center, Colton, USA; 3 Neurosurgery, Riverside University Health System Medical Center, Moreno Valley, USA; 4 Neurosurgery, Desert Regional Medical Center, Palm Springs, USA; 5 Neurosurgery, Arrowhead Regional Medical Center, Colton, USA; 6 Neurosurgery, California University of Science and Medicine, Colton, USA

**Keywords:** altered consciousness, glasgow coma scale (gcs), limitations, neuro-critical care, neuro-surgery, scale composition, traumatic brain injury (tbi)

## Abstract

Traumatic brain injury (TBI) is a prominent cause of long-term disability and death in the United States. The Glasgow Coma Scale (GCS) plays a crucial role in managing TBI by providing a standardized method for assessing severity, monitoring progression, guiding treatment decisions, predicting outcomes, facilitating communication, and supporting research and quality improvement efforts. The scale offers a practical approach to assessing the impairment of consciousness in response to specific stimuli. However, to date, there is a paucity of literature discussing the limitations of the GCS. In this narrative review, we have analyzed seven studies published between 2009 and 2024 in hopes of highlighting some of the limitations, such as potential subjectivity in scoring, inability to assess certain brainstem reflexes, and the need for supplementary assessments for specific neurological conditions. After reviewing literature from the past 15 years, several limitations of the GCS become apparent, including its failure to incorporate brainstem reflexes with limitations arising due to sedation and intubation of the patient. Moreover, the GCS consists of three sub-scales that are summed and assumed to carry equal weight. This can result in a loss of information as it is possible to achieve identical GCS scores through various combinations. Awareness of the limitations of the GCS can be crucial for clinicians when making decisions in specific scenarios, while also encouraging consideration of potential improvements to the scale.

## Introduction and background

The Glasgow Coma Scale (GCS) was introduced in 1974 at the University of Glasgow by neurosurgery professors Dr. Graham Teasdale and Dr. Bryan Jennett to assess the extent of neurological dysfunction in acute medical and trauma patients. It should be noted that the GCS can prove valuable in evaluating any condition resulting in altered consciousness [1]. Since its inception, the GCS has emerged as a nearly universal tool for trauma care practitioners to monitor a patient’s neurologic course during their hospital stay and to anticipate a patient’s future neurological outcome. Notably, the GCS is used not only by neurospecialists but also by non-specialists such as emergency medical responders, general practitioners, and critical care nurses, due to its simplicity and accessibility. Table 1 highlights the three core aspects of responsiveness assessed by the GCS: eye-opening, motor, and verbal responses. These components, while simple to evaluate, reflect distinct neurological functions.

**Table 1:** The Glasgow Coma Scale GCS of 13-15: Mild head injury GCS of 9-12: Moderate head injury GCS of 3-8: Severe head injury

Score	Eye Opening	Verbal Response	Motor Response
6	-	-	Obeys commands
5	-	Oriented	Localizes pain
4	Spontaneously	Confused	Withdraws from pain
3	To voice	Inappropriate words	Abnormal flexion
2	To pain	Incomprehensible sounds	Abnormal extension
1	No response	No response	No response

Eye-opening, the first component, primarily evaluates the function of the ascending reticular activating system (ARAS) within the brainstem, which plays a critical role in maintaining arousal and wakefulness. A patient’s ability to open their eyes spontaneously suggests an intact ARAS and cerebral hemispheres, whereas the need for stimulation or a lack of eye-opening signifies varying levels of brainstem or cortical impairment.

The motor response component assesses the integrity of both central and peripheral motor pathways. It reflects the functionality of motor cortices, corticospinal tracts, and their interactions with the brainstem and spinal cord. Purposeful movement in response to stimuli demonstrates higher-level cerebral function, while abnormal posturing, such as decerebrate or decorticate responses, indicates significant brain injury or dysfunction at specific levels of the central nervous system.

Verbal responses provide insight into the patient’s ability to generate language, which involves multiple regions of the brain, including the Broca and Wernicke areas, and their connection to the brainstem. Speech coherence and orientation depend on the integrity of these regions as well as adequate oxygenation and metabolic function.

Since its creation for assessing neurological dysfunction, the GCS has found applications beyond this initial purpose. In modern medical practice, it predominantly serves as a tool to predict patient outcomes following head trauma. It has also been incorporated into other scoring systems, such as the Intracerebral Hemorrhage (ICH) score, utilized for prognostication in cases of ICH. Additionally, the GCS is used to guide whether surgical or medical management is needed in conditions like subdural hematoma (SDH), where all patients with acute SDH and a GCS score of less than 9 should undergo intracranial pressure (ICP) monitoring. On the other hand, a patient with a GCS score below 9, SDH thickness less than 10 mm, and midline shift less than 5 mm should consider surgical evacuation if the GCS score decreased by 2 or more points between injury and hospital admission [2].

When treating critically ill patients, communication with families is especially important to allow transparency and understanding by all stakeholders of the severity of the patient’s condition. The term “coma” in the Glasgow Coma Scale may be misleading for laymen when they hear their physician refer to a GCS 8 or less as a coma; accordingly, if patients with GCS of 9 through 15 are not actually in a coma, perhaps the score would more ideally be referred to as the Glasgow Consciousness Scale, vs. the Glasgow Coma Scale? This paper aims to explore the limitations of the GCS, highlighting its challenges in clinical practice and suggesting potential modifications to improve its utility and accuracy.

## Review

Methods

A systematic literature review was conducted to identify relevant studies published without temporal restrictions. The initial search was carried out in the PubMed database using the search term "Limitations of the Glasgow Coma Scale," yielding 1,445 potential articles for inclusion.

To refine the scope of our research, we applied additional filters to include articles published within the past 15 years, specifically from 2009 to 2024, using the same search query. This approach resulted in 1,064 articles. Following this, we excluded one article due to being written in a non-English language and three additional articles due to their requirement for subscription-based access. As a result, 1,060 articles were assessed for eligibility. Of these, five specifically examined the limitations of the GCS in patients with traumatic brain injury (TBI).

The inclusion criteria for this review consisted of studies that focused on the GCS and its limitations in clinical or research settings, were published in English, examined patients with TBI, and were published between 2009 and 2024.

**Figure 1:**
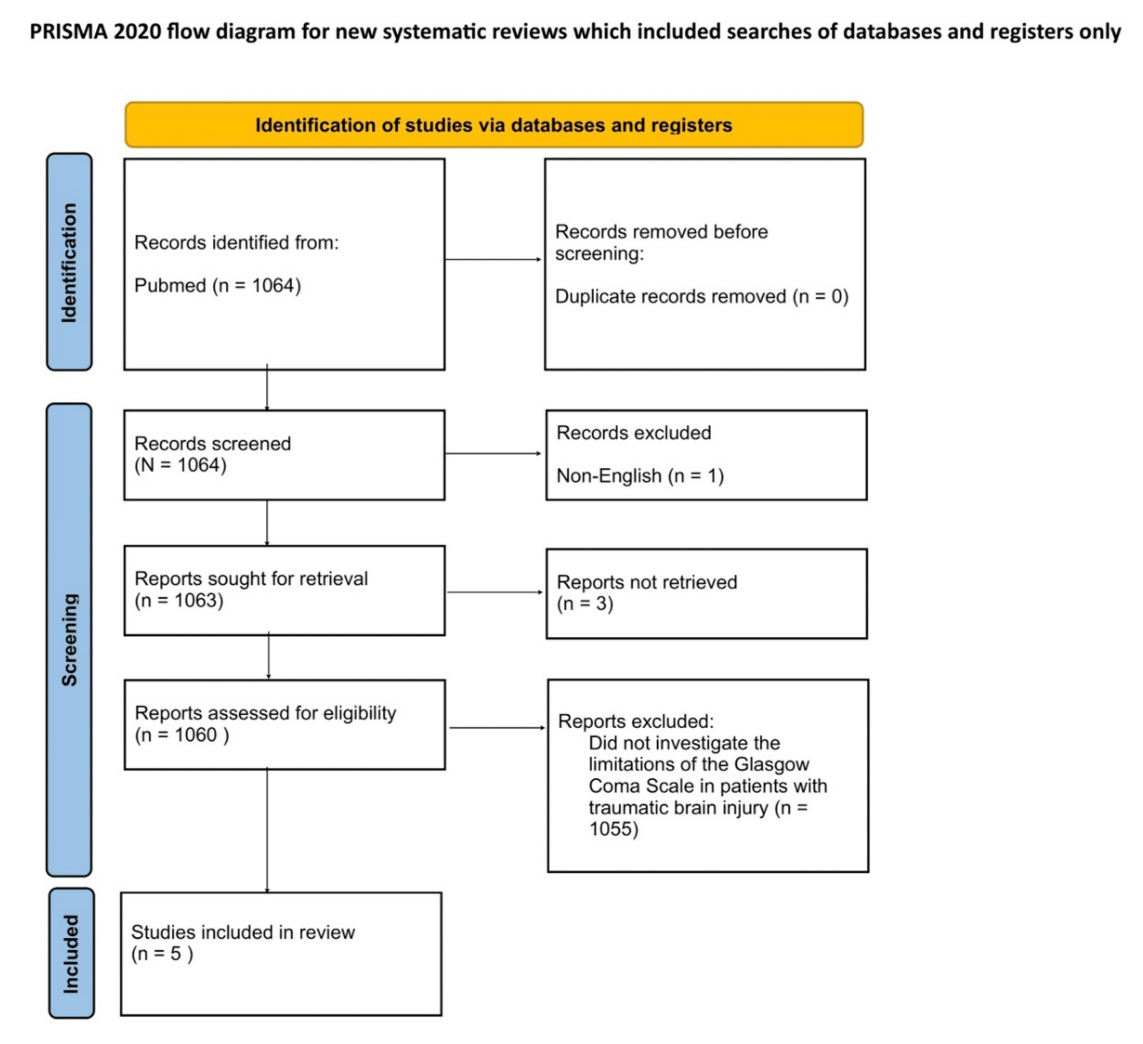
PRISMA flow diagram PRISMA: Preferred Reporting Items for Systematic Reviews and Meta-Analyses

Exclusion criteria included articles that were behind a paywall or required subscription access, studies that did not directly address the limitations of the GCS, particularly in relation to patients with neurological injury, and articles not published in English (Figure 1).

Trials and outcomes

Ghaffarpasand et al. examined the limitations and reliability of the GCS in pediatric patients with TBI, a leading cause of pediatric emergency visits (Table 2) [3]. While children generally have better TBI outcomes than adults, factors such as incomplete myelination in younger children and developmental differences complicate prognostication. The GCS, though widely used, can be inconclusive in pediatric populations due to challenges like altered consciousness, limited comprehension of commands, and hypoxic-ischemic insults. To address this, a modified scoring system has been proposed, setting a GCS score of 5 as the threshold for severe TBI. With this adjustment, children scoring 3-5 face higher mortality, while those above 5 typically achieve better outcomes. Despite its limitations, the GCS remains the most reliable predictor of pediatric TBI when tailored to this population.

**Table 2:** Summary of objectives and outcomes in studies addressing limitations of the Glasgow Coma Scale

Author, year	Objective	Outcome
Ghaffarpasand et al., 2013 [3]	Examine the limitations and reliability of the Glasgow Coma Scale (GCS) in pediatric patients with traumatic brain injury (TBI).	It is more difficult to predict the outcome of a TBI in pediatric populations. This is because the scoring system is based on consciousness and patients’ understanding of the orders and commands, which may not be applicable in certain pediatric cases. Thus, the threshold for neurophysiologic dysfunction should be decreased in the pediatric population. It has been suggested that the cut-off value should be set at 5 as a severe TBI to better assess a more precise outcome.
Reith et al., 2016 [4]	Investigate the variations in the application and interpretation of the GCS among healthcare professionals worldwide	Major differences were found regarding the type of stimulus applied. Strategies for reporting the GCS varied greatly and 35% of study participants limited the reporting to a summary score. This current study found that 30% of responders still use the original motor scale, totaling the GCS to 14 instead of 15. This study highlighted the need for continued education and standardization of the use of the GCS to improve the reliability of assessments of patients who have suffered from TBI
Rozenfeld et al., 2020 [5]	Evaluate how age influences the reliability and effectiveness of the GCS.	The likelihood of having a GCS score below normal rose with younger age and higher injury severity. The specific connection between age and GCS scores remained ambiguous, though several proposed explanations included variations in injury mechanisms across age groups, differing levels of pre-injury alcohol consumption, a higher proportion of elderly females, and potential delays in response to injury due to anatomical factors. Screening protocols for TBI patients should place more emphasis on both the age of adult patients as well as the mechanism of injury.
DiGiorgio et al., 2020 [6]	Investigate how drug and alcohol intoxication affect the assessment of GCS in patients with TBI.	The average change in GCS score showed a notable increase among patients with impaired states compared to those who tested negative for intoxicants. Among 187 patients with an initial GCS score of 3, 150 had positive toxicology screens. The impaired group exhibited a significantly higher change in GCS (2.75 ± 2.7) compared to the unimpaired group (1.19 ± 1.8). To improve utility of the GCS, screening for substances is recommended, and measures should be taken to either reverse their effects or allow them to dissipate before recording GCS scores for benchmarking or quality reporting purposes.
Bodien et al., 2021 [7]	Investigate the relationship between GCS total scores and level of consciousness in adult subjects assessed with the GCS.	Published criteria of multiple disorders of consciousness (DoC) were studied, including coma, unresponsive wakefulness syndrome, minimally conscious state, and post-traumatic confusion state. All GCS total scores between 4-14 were associated with more than one disorder of consciousness diagnosis, with the greatest variability was observed for scores of 7-11. Further, a wide range of total scores were associated with identical DoC diagnoses. The GCS score does not accurately depict level of consciousness as shown in published DoC diagnostic criteria. Subscale score analysis becomes particularly critical when dealing with GCS total scores of 8 or lower due to the risk of misdiagnosing coma.

Reith et al. examined the lack of standardization in GCS assessments, highlighting concerns about reliability and consistency [4]. Surveys conducted online and during neurosurgical training, involving 616 participants from 48 countries, revealed major variations in stimuli used for non-responsive patients (e.g., nail bed pressure, trapezius pinch) and reporting methods, with 35% of respondents using only a summary score. Additionally, 30% of participants reported using the original five-step motor scale, resulting in a GCS total of 14 rather than 15, further underscoring inconsistencies. Reith et al. emphasized the importance of reporting the full GCS for individual assessments and reserving the sum score for group-level comparisons. The study underscores the need for standardized guidelines and ongoing education to improve the reliability of GCS use in TBI assessments.

Rozenfeld et al. conducted a retrospective study analyzing how age influences GCS scores in isolated TBI patients using data from 18,534 cases in the Israeli National Trauma Registry (1997-2017) [5]. Patients were grouped into four age brackets (20-44, 45-64, 65-74, and 75+ years), with factors like gender, Abbreviated Injury Scores (AIS), and injury circumstances considered. Two-thirds of patients were male, and 60% were aged 65 or older. GCS scores were significantly influenced by age, injury severity, and AIS, with younger patients and those with severe injuries more likely to have lower GCS scores. Interestingly, over twice as many patients aged 75+ had a GCS of 15 compared to those aged 20-44. The link between age and GCS scores was attributed to factors like injury mechanisms, pre-injury alcohol use, and anatomical differences. Rozenfeld et al. recommended tailoring TBI screening protocols to account for patient age and injury mechanisms.

DiGiorgio et al. examined how drug and alcohol intoxication impact GCS assessment in TBI patients using trauma registry data (2013-2017) [6]. Among 468 patients with blunt head trauma, toxicology screens revealed no substances in 46.4%, while 22.4% tested positive for alcohol or marijuana, 20.1% for benzodiazepines, 10.3% for opiates, and 8.8% for cocaine. Of 187 patients with an initial GCS of 3, 150 had positive toxicology results. Intoxicated patients showed greater GCS changes (2.75 ± 2.7) compared to those without substances (1.19 ± 1.8). The study concluded that intoxication complicates GCS assessment and recommended screening and addressing intoxicants before recording scores for accurate benchmarking.

Bodien et al. investigated the relationship between GCS scores and levels of consciousness in 2,455 adults with disorders of consciousness (DoC) [7]. They found that GCS scores from 4 to 14 correlated with multiple DoC diagnoses, with the most variability seen in scores of 7-11. Surprisingly, no patients with GCS scores of 7-8 were diagnosed with coma, challenging the use of a GCS score of 8 as a threshold for defining "coma." The study emphasized that clinicians should consider GCS subscale scores and behavioral profiles, especially for scores ≤8, to avoid misdiagnoses.

Discussion

The included studies were selected based on their methodological rigor and relevance to the topic. Ghaffarpasand et al. provided a foundational analysis of the GCS' limitations in accurately predicting outcomes for patients with severe TBIs, employing a robust dataset and clear statistical analysis. Reith et al. contributed a comprehensive meta-analysis, which synthesized data from various studies to identify systemic inconsistencies and biases in the application of the GCS, thereby offering valuable insights into its practical limitations.

Rozenfeld et al. took an innovative approach by exploring specific patient populations and emphasizing how demographic factors, such as age and comorbidities, could influence GCS scores. Similarly, DiGiorgio et al. focused on the predictive accuracy of GCS in guiding clinical decision-making, highlighting its limitations in complex, real-world scenarios. Finally, Bodien et al. offered a nuanced perspective on the scale, suggesting revisions to improve its reliability and applicability in contemporary medical practice.

The GCS' eye response assessment focuses on eye-opening but neglects key neurological indicators such as pupil size and light reactivity, which are critical for identifying conditions like uncal herniation. Pupillary dilation in TBI may signal a neurological emergency, while absent pupil reactivity often indicates a poor prognosis. The verbal response assessment can also be problematic. Language barriers, damage to Broca’s area, physical impediments like tongue or larynx injury, or intubation can lead to misinterpretation of responses. Additionally, motor function may be overrepresented, as fully alert patients with "locked-in syndrome" may be unable to follow commands. These disparities highlight the unequal weighting of GCS subscales, which can result in patients with the same total GCS score being categorized with vastly different mortality risks.

Painful stimuli used in the Best Eye and Best Motor Response assessments can complicate scoring, especially in polytrauma patients with spinal cord injuries [4]. The GCS also struggles to reflect neurological burden accurately. For instance, a quadriplegic patient and a neurologically intact individual could both score 15, while a comatose patient, one in silent status epilepticus, or even a deceased person may all score 3. These limitations reveal contradictions in how the scale interprets neurological function. As GCS scoring evolves, it is crucial to recognize its shortcomings in assessing both isolated head trauma and overall neurological burden.

Executive summary

The GCS is a widely used tool for assessing neurological function, but it has notable limitations in reliability, especially in patients with aphasia, intubation, or complex injuries. Modifications to the GCS, such as incorporating pupillary reactivity or using a "short-form" version, have shown promise in improving its predictive accuracy. However, identical GCS scores can represent varying neurological states, highlighting the need for more refined scoring systems tailored to specialized populations. Future research should prioritize validating these modifications and exploring advanced tools and biomarkers to enhance the GCS’s clinical utility.

Future directions

Despite the GCS limitations in patients with aphasia, its simplicity and widespread use make it invaluable in neurosurgical care. To improve its prognostic utility, clinicians have proposed a modification incorporating pupillary reactivity, where each non-reactive pupil subtracts one point from the total GCS score [8]. This adjustment, validated using CRASH and IMPACT database data, aims to enhance outcome predictions in neurotrauma patients.

In practice, this modification could be easily integrated into routine trauma and ICU assessments. Standardized training for trauma teams and neurosurgical staff could ensure its consistent application. Notably, this approach may address discrepancies in outcomes, such as cases where patients with a GCS of 3, due to a postictal state, have better prognoses than those with severe posturing and a GCS of 4.

Further research could validate this modification through large, multicenter studies focusing on long-term outcomes like recovery, disability, and mortality. These studies should account for factors such as age, pre-existing conditions, and injury severity.

The GCS also faces limitations in patients with aphasia. For example, even small ICHs in speech centers can depress GCS scores more than warranted, failing to reflect the true severity of cortical injury. Aphasic patients often face complex challenges in communication and processing information, leading to disproportionately low motor and verbal scores, especially with non-dominant hemisphere injuries.

Future research could explore GCS adjustments for aphasic patients, such as emphasizing purposeful actions to refine motor scoring. The short-form GCS, which evaluates only motor and pupillary responses, has shown similar predictive value to the full scale in stroke patients [9]. Comparative studies on the short-form GCS, Neurological Pupil Index, and biomarkers like glial fibrillary acidic protein (GFAP) [10] could lead to a more accurate scale tailored to specialized populations, including aphasic patients.

## Conclusions

The GCS has been in use for 47 years and has been greatly utilized to assist in determining the severity of dysfunction following TBI or any condition leading to impaired consciousness. It is one of the most common tools used by trauma care providers as it allows for the gradation of head injury by using observations rather than invasive techniques. However, the GCS has often been criticized for its failure to incorporate brainstem reflexes and the limitations it has due to sedation and intubation of the patient. Also, there have been instances of the GCS demonstrating very low interrater reliability. There has been recent research suggesting modifications to the GCS such as subtracting a point for each non-reactive pupil. Moreover, the GCS is limited in its ability to evaluate patients with aphasia, or patients with concomitant head and spinal cord injury. As previously mentioned, a comatose patient receives a GCS score of 3, which coincidentally matches the GCS score assigned to a deceased individual.

The GCS is limited in assessing a patient’s neurological burden, thus limiting its use in prognostication. There have been attempts to utilize a “short-form” GCS which utilizes motor and pupillary responses while neglecting verbal response in stroke patients. Although the GCS is a great tool that is widely used by trauma care practitioners, there is room for modifications that would improve its purpose, which is to determine the severity of dysfunction. Improving the GCS would ultimately lead to better patient care across all hospitals that utilize this scale, but perhaps the easiest step would be to rename the GCS as the Glasgow Consciousness Scale, reflecting the fact that a GCS of 9 or more is not defined as coma.
